# Prevalence of professional fulfillment, and intention to leave and its risk factors among academic physicians at Ain Shams University, Egypt

**DOI:** 10.1186/s12909-026-08888-3

**Published:** 2026-04-06

**Authors:** Dina Abbas Mohamed AbdelRahman, Manar Mohamed Ellaban

**Affiliations:** Department of Community, Environmental and Occupational Medicine, Faculty of Medicine, Ain Shams University, Cairo, Egypt

**Keywords:** Burnout, Professional, Intention to Leave, Physicians, Universities, Egypt

## Abstract

**Background:**

Physician turnover is a big burden on health care system and is a real risk on it. Burnout and dissatisfaction among physicians increasingly threaten workforce stability, turnover intentions, and healthcare quality, particularly in academic settings. This study measured prevalence of burnout, professional fulfillment (PF), and intention to leave (ITL) among academic physicians at Ain Shams University and identifying ITL predictors.

**Methodology:**

In this cross-sectional study (*n* = 260), data on sociodemographic, Professional Fulfillment Index (PFI) subscales (burnout/PF), single-item ITL, and contributing factors were collected via electronic survey. Bivariate (t-tests/Chi-square) and multivariate stepwise logistic regression (SPSS v24; *p* < 0.05) analyses identified predictors.

**Results:**

PF was low (3.8%), burnout high (70%), and moderate-to-high ITL prevalent (63%). Multivariate analysis showed low PF (aOR = 0.04, 95% CI: 0.003–0.45, *p* = 0.007) and burnout (aOR = 2.04, 95% CI: 1.4–2.9, *p* = 0.001) as independent ITL predictors. Common factors included work pressures, poor alignment, and lack of recognition.

**Conclusions:**

Substantial burnout and low PF among academic physicians associate with elevated ITL after adjustment, signaling workforce risks. Institutional efforts to improve work conditions and support are warranted, with longitudinal studies needed to assess causality.

## Introduction

Physician turnover threatens healthcare system stability worldwide. Physicians form the cornerstone of healthcare systems. In recent years, physician migration has increased due to multiple push factors. Intention to leave (ITL) is defined as a moderate or strong intention to quit one’s institution within two years [1]. While Professional fulfillment (PF) encompasses job satisfaction, accomplishment, self-worth, and self-efficacy [2]. The stability of the staff and the standard of care are at risk since ITL is a predictor of real turnover [3]. Global physician burnout affects 30–35% [4], rising to 70% in Egyptian public sectors [5]. ITL prevalence reaches 32.6% in US academics [1], yet Egyptian academic physician data remains scarce.

The healthcare sector, especially at academic institutions, faces substantial challenges that affect the professional paths and general well-being of doctors. Academic doctors face particular challenges because they work at the nexus of patient care, research, and teaching [1]. Risk factors for ITL include burnout and low PF (validated Professional fulfillment Index (PFI) subscales), poor organizational support, leadership, peer support, values misalignment, and work-personal conflicts [1, 5].

In Egypt, resource shortages, high patient loads, and healthcare reforms exacerbate these challenges [6]. These pressures underscore the need to identify retention factors [7]. Academic physicians in private universities, both globally and in Egypt, report higher ITL compared to those in governmental public universities, largely due to job insecurity, weaker institutional support, and limited career development opportunities. Public universities benefit from government backing, tenure systems, and structured promotion pathways, which reduce turnover intention [8].

Since Ain Shams University (ASU) is essential to Egypt’s medical education and healthcare system, it provides an ideal study setting. It is anticipated that the results will guide focused actions that improve job satisfaction and lower ITL, advancing Egypt’s larger objectives for healthcare reform [9, 10].

Despite regional burnout studies [5, 7], limited literature examines ITL predictors among Egyptian public university faculty, justifying this ASU investigation. This study measures ITL prevalence and predictors among ASU academic physicians to inform retention strategies. as a step to improve the status of physicians in Egypt and improve the health care system. These findings will guide institutional strategies to mitigate burnout, enhance fulfillment, and reduce ITL in Egyptian public universities like ASU.

*Aim*: To measure the prevalence of burnout, PF, and ITL clinical and non-clinical academic physicians at ASU, Egypt, and to identify key predictors of ITL.

*Research Questions*: What is ITL prevalence among ASU academic physicians? Are burnout/PF independent ITL predictors after adjustment? 

*Hypotheses*: H1: Higher burnout predicts greater ITL (aOR>1.0). H2: Higher PF protects against ITL (aOR<1.0). 

## Methods

*Design*: Cross-sectional survey conducted between April and September 2024.

*Setting*: ASU Faculty of Medicine, Cairo, Egypt.

*Participants*: Academic physicians from clinical departments (internal medicine and subspecialties [48.1% of clinical participants], surgery and subspecialties [6.1%], anesthesia and ICU [3.1%]) and non-clinical departments (anatomy, physiology, public health) participated.

Inclusion Criteria: Academic physicians (demonstrators to professors) employed ≥1 year at ASU Faculty of Medicine.

Exclusion Criteria: Administrative and non-academic staff, visiting faculty members, and non-responders.

*Sampling*: Convenience sampling from 320 eligible ASU academic physicians; 260 provided complete responses to the electronic survey that was distributed via the official university email accounts (81.25% response rate; 60 non-responders). Administrative records confirmed no significant demographic differences between responders and non-responders.

### Variables

*Primary: ITL*; A single-item Likert scale (0–4) measured ITL. Moderate-to-high ITL was indicated by ≥ 2) [1].

*Secondary: PF*; a 6-item measure of PF normalized to a 0–10 scale and evaluated on a 5-point Likert scale (0–4). PF was indicated by a score of ≥ 7.5 [11]; Burnout (The 10-item PFI burnout scale quantifies burnout, assessing interpersonal disengagement and occupational weariness. Scores were normalized to 0–10; burnout was indicated by ≥ 3.325) [12]. PF and burnout are distinct subscales from single PFI instrument. Original English versions used (development language; Trockel et al. [11]). PFI demonstrates strong internal consistency (α = 0.86–0.93) and validity across 20 + countries. Despite no Egypt-specific validation available, English deemed appropriate given medical academia’s predominant English usage for research instruments.

*Covariates: Sociodemographic* (age, sex, academic position, years at ASU, specialty, department, weekly work hours); *work/non-work factors; personal-organizational alignment; self-compassion; perceived gratitude.*

*Data Sources/Measurement (Instruments)*: An anonymous, structured electronic questionnaire adapted from the literature was used [1, 11, 12]. The electronic questionnaire was in English language. Informed consent was obtained electronically at the beginning of the survey. The questionnaire included five sections: Sections (I) Sociodemographic/occupational data (age, sex, academic position, years at ASU, specialty [clinical/non-clinical], department, weekly work hours); Section (II): PFI ; Section (III) PFI burnout; Section (IV) ITL; Section(V) Work/non-work factors, self-compassion, gratitude, values alignment.

*Bias*: Convenience sampling and online email distribution may introduce selection and digital access biases. Self-reported data is susceptible to social desirability bias. These risks were mitigated by: (1) universal university email access for all faculty; (2) high response rate (81.25%, 260/320); (3) no significant demographic differences between responders/non-responders per administrative records.

*Study Size*: The target sample size of 260 was determined a priori using OpenEpi v3.01, with anticipated moderate-to-high ITL`` prevalence of 32.6% reported by Ligibel et al. (2023), 8% margin of error, 95% confidence level, and design effect of 2.

Sample size calculation formula: n = [Z² × p × (1-p) / d² × N] / [1 + (Z² × p × (1-p) / d² × N)] Where: Z = 1.96 (95% CI) *p* = 0.326 (ITL prevalence, *Ligibel et al. 2023*) d = 0.08 (8% margin of error) *N* = 320 (ASU faculty population) Design effect = 2 (departmental clustering). The initial calculation yielded *n* ≈ 94, which was adjusted for departmental clustering using a design effect of 2 (*n* ≈ 188). To ensure adequate power for subgroup analyses and account for potential nonresponse, we conservatively increased the target sample size to 260 participants. This approach guarantees robust estimates across all study objectives.

*Quantitative variables*: Demographic and occupational characteristics were summarized using descriptive statistics. Continuous variables were shown as means ± standard deviations, whereas categorical variables were shown as frequencies and percentages.

*Statistical Methods*: Data were coded and analyzed using SPSS version 24. Bivariate analyses used independent t-tests for continuous outcomes (PF, burnout scores) between independent groups meeting assumptions (Shapiro-Wilk *p* > 0.05 normality; Levene’s *p* > 0.05 equal variances). Chi-square tests analyzed categorical variables. Binary logistic regression identified independent predictors of: Model 1: ITL (binary: 0 = no, 1 = yes) as dependent variable; Model 2: Moderate-high ITL (binary: 0 = no/low [< 2], 1 = moderate-high [≥ 2]) as dependent variable. Variables with bivariate *p* < 0.2 entered via stepwise forward selection (*p* ≥ 0.10 removed iteratively). Statistical significance: *p* < 0.05. Hierarchical blocks: (1) sociodemographic (2), work factors (3), burnout/PF. Multicollinearity: VIF < 2.5. Model fit: Hosmer-Lemeshow χ²=8.2, *p* = 0.41; c-statistic = 0.78.

## Results

### Participant characteristics (*n* = 260)

This cross-sectional survey included 260 academic physicians (81.25% response from 320 eligible) with a mean age of 33.8 ± 4.9 years; 76.9% were female. Lecturers comprised the largest academic group (40.8%), followed by assistant lecturers (25.8%) and demonstrators (21.9%). Clinical specialties represented 57.3% of participants, with internal medicine and its subspecialties being most common (48.1%), followed by surgery and its subspecialties (6.1%) and anesthesia/ICU (3.1%). Non-clinical specialties comprised 42.7%. The mean self-reported weekly workload was 30.2 ± 23.9 h, and average employment duration was 8.7 ± 5.3 years. Comparing moderate-to-high ITL with no/low ITL groups (*n* = 165 vs. 95) showed no significant differences in sociodemographic variables (all *p* > 0.05), except a borderline non-significant higher proportion of clinical specialists in the moderate-high ITL group (54.5% vs. 42.7%, *p* = 0.07) (Table 1).

**Table 1:** Sociodemographic data among moderate to high ITL and non ITL

Variables			Total(n=260)N (%)	Moderate to high ITL(n=165) (63.5%)N (%)	No and low ITL(n=95) (36.5%)N (%)	*P* value
Age (years): mean ± SD			33.79 ± 4.93	33.76 ± 5.2	33.8 ± 4.6	0.903
Gender	Male		60 (23.1%)	42(25.5%)	18(18.9%)	0.230
	Female		200 (76.9%)	123(74.5%)	77(81.1%)	
Academic position	Professor		5 (1.9%)	3(1.8%)	2 (2.1%)	0.285
	Assistant professor		25 (9.6%)	19 (11.5%)	6 (6.3%)	
	Lecturer		106 (40.8%)	63(38.2%)	43(45.3%)	
	Assistant lecturer		67 (25.8%)	39 (23.6%)	28 (29.5%)	
	Demonstrator		57 (21.9%)	41(24.8%)	16 (16.8%)	
Specialty	Clinical		111 (42.7%)	90(54.5%)	59(62.1%)	0.235
	Nonclinical		149 (57.3%)	75(45.5%	36(37.9%)	
Department	Non-clinical (Academic)		111 (42.7%)	75 (45.5%)	36 (37.9%)	
	Clinical 149 (57.3%)	Internal medicine	125 (48.1%)	71(43%)	54(56.8%)	0.106
		Surgery	16 (6.2%)	12 (7.3%)	4 (4.2%)	
		Anesthesia and ICU	8 (3.1%)	7 (4.2%)	1 (1.1%)	
Years of work at faculty of medicine ASU: mean ± SD			8.7 ± 5.3	8.4 ± 5.1	9.1 ± 5.2	0.331
Work hours per week: mean ± SD			30.2 ± 23.9	32.0 ± 24.9	27.1 ± 22.8	0.113

### Primary outcome: ITL

Moderate-to-high ITL over the next two years affected 63.5% of participants (definitely: 15%; likely: 28.5%; moderate: 20%). Furthermore, 22.7% had already begun planning departure within the previous three months. The distribution of ITL responses across the four categories was statistically significant (*p* = 0.001) (Table 2).

**Table 2:** Prevalence of burnout, professional fulfillment and intention to leave among moderate to high ITL and non ITL

Variables	Total(n=260)N (%)	Moderate to high ITL(n=165)(63.5%)N (%)	No and low ITL(n=95)(36.5%)N (%)	*P* value
Physicians who started in the preparation of leaving their institution within the last 3 month	59 (22.7%.)	58 (35.2%.)	1 (1.1%)	0.001
Frequency of ITL their institution within the next 2 years:	39 (15%)			0.001
Definitely	74 (28.5%)	39 (23.6%)	0 (0%)	
Likely	52 (20%)	74 (44.8%)	0(0%)	
Moderate	95 (36.5%)	52 (31.5%)	0(0%)	
No ITL	165 (63.5%)	0(0%)	95(100%)	
Prevalence of burnout	182 (70%)	118(71.5%)	64(67.4%)	0.574
Prevalence of professional fulfillment	10 (3.8%)	2(1.2%)	8 (8.4%)	0.004
Work related factors	188(72.3%)	116(70.3%)	72(75.8%)	0.341
Non-Work-related factors	72 (27.7%)	49(29.7%)	23(24.2%)	
Work related causes:				
low income related to workload	153 (58.8)	98 (79.7%)	55(80.9%)	0.841
work related stress	130(50%)	65(52.8%)	65(95.6%)	0.001
poor administration	98 (37.7%)	56 (45.5%)	42(61.8%)	0.032
poor interpersonal relationships	84 (32.3%)	50 (40.7%)	34 (50%)	0.213
poor environmental conditions	63 (24.2%)	63 (51.2%)	0 (0%)	0.001
work shift	34 (13.1)	16 (13%)	18 (26.5%)	0.020
Biological hazards:	14 (5.4%)	14 (11.4%)	0 (0%)	0.004
Physical hazards:	7 (2.7%)	7 (5.7%)	0 (0%)	0.045
Chemical hazards	2 (0.8%)	2 (1.6%)	0 (0%)	0.290
Non-Work-related causes:				
Low appreciation (feel un appreciation at work)	30 (41.6%)	25 (15.2%)	0 (0%)	0.0001
Financial issues (low income of doctors in Egypt, seeking money)	26 (36.1%)	30(18.2%)	0 (0%)	0.0001
Family issues	20 (27.7%)	20(12.1%)	0 (0%)	0.0001
Personal issues as (ambition, continue study abroad, motivational issues and travelling love) was 4%	6 (8.3%)	10(6.1%)	0 (0%)	0.014

### Secondary outcomes: PF and burnout

Only 3.8% (*n* = 10) of academic physicians achieved PF, while burnout was highly prevalent, affecting 70% (*n* = 182) of participants. Low PF and high burnout indicate dissatisfaction. Although burnout prevalence did not differ significantly between groups with moderate-high versus no/low ITL (71.5% vs. 67.4%; *p* = 0.574), PF was significantly lower in those with moderate-high ITL (1.2% vs. 8.4%, *p* = 0.004) (Table 2).

### Factors contributing to ITL

Work-related factors were the most frequently cited causes of ITL, reported by 72.3% of participants. These included inadequate income relative to workload (58.8%), work-related stress (50.0%), poor administration (37.7%), and poor interpersonal relationships (32.3%). Other work-related issues comprised poor environmental conditions (24.2%), shift work (13.1%), and exposure to occupational hazards including chemical (0.8%), physical (2.7%), and biological (5.4%) risks. Non-work-related factors affected 27.7% of participants and included lack of recognition (41.6%), financial difficulties (36.1%), family duties (27.7%), and personal motivations such as ambition or desire to study abroad (8.3%). Bivariate analyses indicated significant differences between moderate-high and no/low ITL groups for most work-related factors (e.g., inadequate income, work stress, administration, interpersonal relations) with p values ranging from 0.001 to 0.045, and all non-work-related factors were significantly associated with ITL (Table 2).

### Personal-organizational alignment and psychological measures

Significant personal-organizational misalignment was evident, with 43.5% reporting mismatched organizational goals and personal values, and only 3.5% feeling their opinions were “completely” recognized in administrative decisions. Additionally, 28.8% reported that administration did not recognize their clinical and academic contributions at all. Psychological measures revealed that 44.0% delayed self-care due to time constraints and 31.5% frequently engaged in self-blame after mistakes. Regarding perceived gratitude from colleagues, 40.4% felt “somewhat” valued while 14.2% felt no gratitude at all. However, gratitude scores did not differ significantly between ITL groups (*p* = 0.526) (Table 3).

**Table 3:** Personal-organizational values alignment, self- valuation (self-compassion and Perceived gratitude moderate to high ITL and non ITL

Variables	No (%)(n=260)	Moderate to high ITL(n=165) (63.5%)N (%)	No and low ITL(n=95)(36.5%)N (%)	*P* value
personal-organizational values alignment:				
My input is valued in important administrative decisions:				
Not at all true	93 (35.8%)	67(40.6%)	26(27.4%)	0.088
Somewhat true	92 (35.4%)	58(35.2%)	34(35.8%)	
Moderate	57 (21.9%)	28(17%)	29(30.5%)	
Very true	9 (3.5%)	6 (3.6%)	3 (3.2%)	
Completely true	9 (3.5%)	6 (3.6%)	3 (3.2%)	
Our organizational goals and values fit well with my goals and values:				
Not at all true	69 (26.5%)	49(29.7%)	20(21.1%)	0.467
Somewhat true	113 (43.5%)	71(43%)	42(44.2%)	
Moderate	59 (22.7%)	35(21.2%)	24(25.3%)	
Very true	7 (2.7%)	3 (1.8%)	4 (4.2%)	
Completely true	12 (4.6%)	7 (4.2%)	5 (5.3%)	
Administration values my clinical and academic work:				
Not at all true	75 (28.8%)	51(31.1%)	24(26.1%)	0.914
Somewhat true	89 (34.2%)	57(34.8%)	32(34.8%)	
Moderate	73 (28%)	42(25.6%)	27(29.3%)	
Very true	16 (6.2%)	10(6.1%)	6(6.5%)	
Completely true	7 (2.7%)	4 (2.4%)	3 (3.3%)	
self- valuation(self-compassion):				
When I made a mistake, I felt more self-condemnation than self-encouragement to learn from the experience:				
Never	23 (8.8%)	16 (9.7%)	7 (7.4%)	0.822
Rarely	59 (22.7%)	37(22.4%)	22(23.2%)	
Sometimes	96 (36.9%)	59(35.8%)	37(38.9%)	
Often	51 (19.6%)	35(21.2%)	16(16.8%)	
Always	31 (11.9%)	18(10.9%)	13(13.7%)	
I put off taking care of my own health due to time pressure				
Never	7 (2.7%)	7(4.2%)	0(0%)	0.005
Rarely	46 (17.7%)	25(15.2%)	21(22.1%)	
Sometimes	93 (35.8%)	68(41.2%)	25(26.3%)	
Often	61 (23.5%)	30(18.2%)	31(32.6%)	
Always	53 (20.4%)	35(21.2%)	18(18.9%)	
Perceived gratitude:				
My colleagues and coworkers appreciate the work I do for my patients or my students				
Not at all	37 (14.2%)	26(15.8%)	10(10.5%)	0.526
Little bit	53 (20.4%)	33(20%)	22(23.2%)	
Moderate bit	105(40.4%)	71(43%)	36(37.9%)	
Quite a bit	37 (14.2%)	20(12.1%)	15(15.8%)	
Extremely	28 (10.8%)	15(9.1%)	12(12.6%)	

### Bivariate and multivariate predictors of ITL

Bivariate analyses showed no significant association between ITL and PF (χ²=3.053, *p* = 0.081) or burnout (χ²=0.019, *p* = 0.923). However, work-related factors were significantly associated with ITL (χ²=4.85, *p* = 0.023; OR = 1.6, 95% CI: 1.06–2.6). By comparing moderate-high ITL (score ≥ 2, *n* = 165) vs. no/low ITL (score < 2, *n* = 95),participants exhibited significantly lower PF scores (3.2 ± 1.9 vs. 3.9 ± 1.7; t = 2.97, df = 258, *p* = 0.004) and higher burnout scores (4.5 ± 1.7 vs. 3.9 ± 1.2; t=-3.12, df = 258, *p* = 0.002). Multivariate logistic regression confirmed burnout as an independent predictor (aOR = 2.04; *p* = 0.001), doubling ITL odds. While PF strongly predicted reduced ITL risk (aOR = 0.04; *p* = 0.007). Work-related factors showed a trend toward significance (aOR = 1.25; *p* = 0.074). Other variables, including personal-organizational values, self-valuation, and perceived gratitude did not independently predict ITL (Table 4). Model diagnostics indicated good fit and discrimination (Hosmer-Lemeshow *p* = 0.41; c-statistic = 0.78) (Table 4).

**Table 4:** Bivariate and Multivariate Predictors of Intention to Leave (ITL)

Model 1: Any ITL (n=59 ITL vs n=201 No ITL)						
Predictor	ITL n (%)	No ITLn (%)	χ² (p-value)	OR (95% CI)	aOR (95% CI)	*p*-value
Burnout	41 (69.5)	141 (70.1)	0.019 (0.923)	0.98(0.52-1.85)	—	—
Professional Fulfillment	0 (0.0)	10 (5.0)	3.053 (0.081)	0.15(0.02-1.20)	0.21 (0.02-1.87)	0.080
Work-related factors	36 (61.0)	152 (75.6)	4.85 **(0.023)**	1.60(1.06-2.60)	1.25 (1.07-1.47)	**0.074**
Personal-Organizational Values (Mean ± SD)	2.5 ± 1.7	2.8 ± 2.1	t=1.22(0.258)			
Self-Valuation (Mean ± SD)	5.5 ± 2.1	5.5 ± 2.7	t=0.19(0.848)			
Perceived Gratitude (Mean ± SD)	4.6 ± 2.8	4.9 ± 2.6	t=1.09(0.442)			

Model 2: Moderate-High ITL (score ≥2; n=165 Moderate-High ITL vs n=95 No/Low ITL)						
Predictor	Moderate-High ITL Mean±SD	No/Low ITLMean±SD	t-test (df=258)	OR (95% CI)	aOR (95% CI)	*p*-value
Burnout score	4.5 ± 1.7	3.9 ± 1.2	-3.12 **(p=0.002)**	—	2.04(1.40-2.90)	**0.001**
PF score	3.2 ± 1.9	3.9 ± 1.7	2.97 **(p=0.004)**	—	0.04 (0.003-0.45)	**0.007**
Work-related factors	116 (70.3)	72 (75.8)	χ²=0.906 (0.341)	0.76(0.45-1.28)	—	—
Personal-Organizational Values (Mean ± SD)	2.6 ± 1.9	3.1 ± 2.0	t=0.45 (0.084)			
Self-Valuation (Mean ± SD)	5.4 ± 1.9	5.6 ± 1.5	t=0.92 (0.355)			
Perceived Gratitude (Mean ± SD)	4.6 ± 2.7	5.1 ± 2.6	t=0.73 (0.158)			

Model diagnostics: Hosmer-Lemeshow χ²=8.2 (*p*=0.41; good fit); c-statistic=0.78 (strong discrimination)

Bold = *p*<0.05. aOR=adjusted odds ratio. Model 1: ITL ≥1 vs 0. Model 2: ITL ≥2 vs <2. Stepwise selection (p<0.2 entry)

## Discussion

According to this cross-sectional study, academic physicians at ASU were in a severe distress. Burnout affected 70%, and PF was extremely low at 3.8%. Additionally, 63.5% report moderate-to-high ITL within two years, with 22.7% already planning departure. These results point to a problem in the workforce that jeopardizes academic medicine’s stability in this setting.

Work-related issues were the main causes of ITL. The most common were “work-related stress” (50%) and “low income related to workload” (58.8%). The “push-pull” migration concept, in which professionals are driven overseas by unfavorable local conditions, is consistent with this. This aligns with Jordanian and Egyptian studies on economic migration drivers [1, 13]. Moreover, a scoping review emphasized that governmental universities and hospitals in Low - middle-income countries (LMICs) struggle with retaining physicians due to limited financial resources, lack of incentives, and weak career development policies. Effective retention strategies include structured promotion pathways, fair compensation, and occupational health protections [14].

Different risk factor patterns according to departure stage were identified by our investigation, which is in line with data from Pakistan that indicates economic considerations were the best indicators of migration [15]. Moreover, workplace policy implications were directly tied to employees’ ITL — strong, fair, and supportive policies reduce attrition, while weak or unfair policies increased it [8]. Psychological factors predominated among those with moderate-to-high ITL, with occupational fulfillment being a key protective factor and burnout raising ITL probabilities. This supports the Job Demands-Resources model [16] and research from the Gulf region that shows burnout to be a significant mediator between work-related pressures and ITL [17].

Interestingly, self-compassion, perceived appreciation, and alignment of personal-organizational ideals did not independently predict ITL, indicating that psychological resources alone are not enough to combat overwhelming basic pressures. This contrasts with research conducted in the West, which shows that retention was more strongly predicted by personal-organizational fit [18], underscoring contextual and cultural variations in retention determinants.

This study’s 70% burnout rate was significantly higher than the global average of 30% to 35% [4], which may be a reflection of the particular socioeconomic stresses found in LMICs. Profound deficiencies in academic career incentives are indicated by similarly low PF percentages (3.8%). Physician burnout rates are similarly concerning in regional research, indicating that this might be a geographical issue [19].

Due to their greater patient care responsibilities and emotional labor, clinical physicians reported a considerably higher number of work-related characteristics than their non-clinical counterparts [20]. In line with Nigerian research that shows a significant level of job stress among academic staff, nearly two-thirds of non-clinical professors also mentioned work-related problems, indicating widespread difficulties throughout academic missions [21].

This study has a number of noteworthy strengths that improve the reliability and applicability of its conclusions. Its main focus is on the important but little-researched topic of physician retention in the setting of academic healthcare in Egypt. In order to accurately evaluate important outcomes and enable cross-cultural comparison, the study used verified, globally known instruments from the PFI. Additionally, a thorough picture of the faculty environment was given by the inclusion of academic physicians who are clinical and non-clinical. On the other hand, we need more studies in this topic and its real causes and factors as a step to improve work status and environmental conditions of academic physicians to decrease their ITL which is a real risk on health care system.

Several limitations should be considered when interpreting these results. The cross-sectional design precludes establishing definitive causality. Reverse causality was minimized by chronic burnout/PF stability [9], prospective ITL measurement (next 2 years), and temporal precedence from prior literature. Convenience sampling and online survey administration may introduce selection and digital access biases, potentially limiting generalizability. Self-reported electronic data is susceptible to social desirability and recall biases. As a single-institution study, findings may not generalize beyond ASU’s organizational context. These risks were mitigated by universal faculty email access, high response rate (81.25%), and no demographic differences between responders/non-responders. Future longitudinal multi-center studies using Structural Equation Modeling to test mediation pathways (work factors → burnout/PF → ITL) are recommended.

## Conclusion

Among ASU academic physicians, burnout and low PF independently predicted moderate-to-high ITL. Primary drivers included inadequate remuneration relative to workload and work stress. Institutions must prioritize fair compensation, workload reduction, and recognition programs. Longitudinal, multi-center studies should test these interventions’ impact on retention.

## Data Availability

Upon reasonable request, the corresponding author will provide the data that support the study’s conclusions. In order to preserve participant privacy, data access may be limited due to confidentiality agreements and ethical reasons.

## Abbreviations

ITL: Intention to leave
PF: Professional fulfilment
PFI: Professional fulfilment Index
ASU: Ain Shams University
LMIC: Low - middle-income countries
